# NLRP3 deficiency abrogates silica-induced neutrophil infiltration, pulmonary damage and fibrosis

**DOI:** 10.1186/s12931-025-03192-y

**Published:** 2025-03-21

**Authors:** Maggie Lam, Kristian T. Barry, Christopher J. Hodges, Christopher M. Harpur, James D. H. Ong, Sarah Rosli, Alison C. West, Lovisa Dousha, Paul J. Hertzog, Ashley Mansell, Michelle D. Tate

**Affiliations:** 1 Centre for Innate Immunity and Infectious Disease, Hudson Institute of Medical Research, Clayton, VIC 3168 Australia; 2 Department of Molecular Translational Sciences, Monash University, Clayton, VIC 3800 Australia; 3 Monash Lung, Sleep, Allergy, and Immunology, Monash Medical Centre, Clayton, Melbourne, VIC 3168 Australia; 4 Department of Medicine, Monash University, Clayton, Melbourne, VIC 3168 Australia; 5 Department of Microbiology, Anatomy, Physiology, and Pharmacology, La Trobe University, Bundoora, VIC 3086 Australia

**Keywords:** Silicosis, NLRP3 inflammasome, Mouse model, Inflammation, Fibrosis

## Abstract

**Background:**

Silicosis is a progressive and often fatal occupational lung disease. The NLRP3 inflammasome is an innate immune sensor that is activated by silica. Accumulating evidence has implicated a role for NLRP3 in silicosis pathogenesis. In this study, we mechanistically elucidated the contribution of NLRP3 to silica-induced pulmonary disease.

**Methods:**

The in vivo role of NLRP3 was investigated following intranasal delivery of 2 mg of silica or diluent alone to wildtype, NLRP3 reporter, and NLRP3-deficient mice. Protein expression, inflammation, and histopathology were analyzed in the lung.

**Results:**

Intranasal administration of silica recapitulated the key pathological features of human silicosis, including nonresolving inflammation, the formation of silicotic nodules, and diffuse lung fibrosis. A reporter mouse placed under the native NLRP3 promoter revealed silica rapidly upregulated NLRP3 expression throughout the lung. NLRP3-deficient mice displayed marked early reductions in silica-induced IL-1β and IL-18 levels in the airways. Additionally, NLRP3 deficiency impaired the rapid infiltration of conventional Siglec-F^−^ and fibrotic Siglec-F^+^ neutrophils, which correlated with reduced levels of neutrophil elastase. Deficiency in acute NLRP3-mediated inflammation correlated with significantly reduced pulmonary transforming growth factor beta and alpha smooth muscle actin expression, tissue damage, and fibrosis in the chronic phase of disease progression. Importantly, this included reduced silicotic nodule size and cellularity.

**Conclusions:**

These findings highlight a major detrimental role for the NLRP3 inflammasome in driving silica-induced pulmonary neutrophil infiltration, TGFβ-mediated myofibroblast activation, tissue damage, and fibrosis.

**Supplementary information:**

The online version contains supplementary material available at 10.1186/s12931-025-03192-y.

## Introduction

Inhalation of crystalline quartz silica can lead to the development of silicosis, a devastating lung disease characterized by nonresolving inflammation, tissue damage, and fibrosis [1]. Despite the implementation of safety standards in industries including mining, manufacturing, and construction, silicosis remains a prevalent global burden [2]. The rapid deterioration of patients following a silicosis diagnosis, including some as young as 25 years old [3], has raised significant public health concerns. This has prompted revisions to global workplace safety regulations. However, the standard for acceptable respirable crystalline silica exposure in India remains at three to six times greater than in countries such as the United States of America and Australia [4]. In response to this growing health crisis, as of July 2024, Australia mandated the compulsory reporting of silicosis cases and implemented a ban on the manufacturing, supply, and use of engineered stone benchtops, which can be composed of up to 95% of crystalline silica [5]. The Global Programme for Silicosis Elimination, established by the International Labour Organisation and the World Health Organisation, aims to eliminate silicosis worldwide by 2030 [6]. Despite the establishment of global goals and initiatives, there are currently no effective treatments for silicosis due to its irreversible nature, with lung transplantation being the only viable option. Management of the disease is largely supportive, with a focus on alleviating symptoms and preventing further silica exposure [7].

Acute silicosis develops within a few years of exposure to extremely high amounts of silica dust and often progresses to secondary pulmonary alveolar proteinosis [8]. In patients with acute silicosis, lungs show severe alveolitis, interstitial inflammation, and the formation of small nodules, which are characteristic features [9, 10]. The inhalation of silica dust initiates a cycle of persistent inflammation and chronic fibrosis in advanced stages of silicosis, which leads to respiratory failure and, in most cases, is fatal. Silica particles are engulfed by resident alveolar macrophages, leading to the activation of downstream inflammatory pathways [1]. The accumulation of silica within these cells impairs their ability to clear the particles, resulting in the exacerbation and preservation of inflammatory responses. This chronic response promotes the formation of silicotic nodules, a key hallmark feature of severe silicosis [11, 12]. Silicotic nodules are initially composed of aggregates of inflammatory cells but become more fibrotic as the disease progresses, eventually forming dense whorls of collagen fibers. Repeated tissue injury caused by silica exposure leads to the expansion and eventual coalescence of silicotic nodules to establish areas of progressive massive fibrosis.

The NLRP3 (nucleotide-binding domain and leucine-rich repeat pyrin domain containing 3) inflammasome is a multiprotein unit responsible for the detection of various cellular stress signals, including environmental irritants such as silica dust [1, 13]. Specifically, silica has been shown to cause lysosomal damage and rupture and mitochondrial dysfunction, which can be sensed by NLRP3 [14]. Interestingly, nearly free silanols (NFS) found on the surface of silica particles can damage phagolysosomes, induce NLRP3-dependent proinflammatory responses, and drive toxicity [15, 16]. Upon activation, NLRP3 interacts with the adaptor protein ASC (apoptosis-associated speck-like protein containing CARD domain) to recruit the cysteine protease caspase-1 to form an oligomeric protein complex [1, 17]. Autocatalysis of caspase-1 subsequently promotes enzymatic maturation of pro-IL-1β and pro-IL-18 to their bioactive forms, IL-1β and IL-18, respectively. Silica-induced pyroptosis, a lytic form of cell death, also involves NLRP3 inflammasome-mediated cleavage of the pore-forming effector gasdermin D (GSDMD) by caspase-1 [18]. Insertion of the active N-terminal of GSDMD into the plasma membrane results in the formation of pores to facilitate the release of potent pro-inflammatory cytokines IL-1β and IL-18 [19, 20]. Eventual plasma membrane rupture promotes the release of cellular contents, including damage-associated molecular patterns (DAMPs), thereby creating a positive feedback inflammation loop [21, 22]. It has also been proposed that silica particles may be released during cell lysis and re-ingested by surrounding cells in a cyclical process [23].

Animal models have been used to recapitulate the major features of human silicosis [24, 25]. A limited number of studies have implicated an in vivo role for NLRP3 in silicosis, with gene-deficient mice displaying reduced gross tissue pathology, including inflammation and fibrosis at 2–3 months [26, 27]. However, NLRP3-dependent responses in the lung are not currently well characterized in the context of silicosis, and further work is required to mechanistically establish the significance of NLRP3 in the pathogenesis of silicosis.

In this study, we investigated a role for NLRP3 in acute and chronic silicosis to provide clearer insights into pathogenesis and the potential of NLRP3 as a therapeutic target. Here, we demonstrated that intranasal administration of 2 mg of silica induces the key pathological hallmarks of silicosis seen in humans, including the formation of silicotic nodules. Using a novel reporter mouse, we identified that silica delivery upregulated NLRP3 expression throughout the lung, particularly in the alveoli at day 3. Notably, NLRP3 deficiency significantly reduced early IL-1β and IL-18 responses in the airways. Additionally, NLRP3 deficiency impaired the infiltration of classical Siglec-F^−^ neutrophils into the airways, as well as a unique population of Siglec-F^+^ neutrophils, which correlated with reduced neutrophil elastase activity. Early NLRP3-mediated inflammation preceded later-stage lung fibrosis marked by collagen deposition and the formation of silicotic nodules. Lastly, mice deficient in NLRP3 displayed reduced fibrosis at day 28 following silica delivery. Suggestive of diminished transforming growth factor β (TGFβ)-mediated myofibroblast activation, NLRP3 deficiency reduced expression of alpha smooth muscle actin (α-SMA) and mature TGFβ in the lung. Importantly, global NLRP3 deletion led to reduced silicotic nodule size and cellularity at day 28. Together, these findings underscore a critical detrimental role for the NLRP3 inflammasome in driving silica-induced inflammation and unresolving pulmonary damage and fibrosis.

## Methods

### Animals

Wildtype, *Nlrp3*^−/−^ and reporter NLRP3-CHCI (mCitrine-NLRP3-mCherry) C57Bl/6J mice (male and female, 6–14 weeks) were housed in specific pathogen-free conditions at the Monash Health Translational Precinct (Clayton, Victoria, Australia). NLRP3-CHCI reporter mice on a C57BL/6J background were generated by gene trap methodology [28, 29] in mouse embryonic stem cells with a vector containing the NLRP3 gene flanked by mCitrine and mCherry (hence called NLRP3-CHCI– i.e., mCitrine-NLRP3-mCherry).

### Mouse model of silica-induced pulmonary disease

Natural, fine-ground silica (Min-U-Sil-5, > 99% purity and particle size of ≤ 5 μm) was kindly provided by U.S. Silica (Katy, USA). Mice were lightly anesthetized with isoflurane and intranasally administered 2 mg of silica in 50 µL phosphate buffered saline (PBS), as previously described [30]. Control mice received PBS alone.

Mice were weighed 3 times a week, and euthanized mice were sacrificed via intraperitoneal injection of sodium pentobarbital. Bronchoalveolar lavage (BAL) was immediately obtained by flushing the lung 3 times with 1 mL of PBS. BAL fluid was collected following centrifugation for cytokine analysis, and BAL cell pellets were analyzed by cytospin or flow cytometry. Lung tissues, post-BAL collection, were removed and immediately frozen in liquid nitrogen for immunoblot analysis of protein expression. Additionally, in a separate cohort of mice, whole lung tissues were formalin-fixed and inflated with 10% neutral buffered formalin (NBF) for histological analysis. Mice were randomly assigned to experimental groups where possible. All procedures were approved by the Hudson Animal Ethics Committee (Application MMCB/2021/18), which adheres to the Australian Code of Practice for the Care and Use of Animals for Scientific Purposes.

### Visualization of BAL cells and silica particles using microscopy

BAL was centrifuged, and the pellet was resuspended in PBS. BAL was then cytocentrifuged onto glass microscope slides (ThermoFisher Shandon Cytospin CytoCentrifuge), fixed, and stained with hematoxylin and eosin (H&E). Five fields of view (FOV) per slide were viewed on an Olympus BX60 microscope (Olympus, Japan) and photographed at 400x magnification with an Olympus DP74 color camera using Olympus cellSens Dimension software. Macrophages/monocytes, lymphocytes, neutrophils, and eosinophils were identified by their distinct nuclear morphologies. The same FOV was also visualized with a polarizer attachment, enabling the detection of birefringent silica particles [30].

To visualize silica in lung tissue sections, unstained lung sections were dewaxed and rehydrated. A customized chamber was built around the lung sample using reusable adhesive putty and filled with distilled water. Imaging was performed on an Olympus FV-MPERS upright multiphoton microscope fitted with an InSight x3 laser, using a tunable laser line tuned to 900 nm. The system was equipped with an Olympus 25x, 1.05 NA water immersion objective lens to ensure optimal resolution and depth penetration, and the laser power used was 100.

### Immunoblot analysis of protein expression in lung tissues and BAL fluids

BAL was collected, and 1 mL of fluid from each mouse was concentrated using Strataclean resin (Agilent Technologies). Remaining lung tissue (i.e., post-BAL collection) was homogenized in 5x SDS lysis buffer (250 mM Tris-HCl (pH 6.8), 10% (w/v) SDS, 20% (v/v) glycerol, supplemented with cOmplete™ Protease Inhibitor (Roche, Basel, Switzerland). Lung protein concentrations were measured using a colorimetric assay to ensure equal protein loading (Bio-Rad DC Protein Assay, Bio-Rad). Lung lysates and protein in BAL fluid were separated by 4–12% SDS-PAGE (Life Technologies) and transferred to PVDF membrane (Merck Millipore). Membranes were blocked with 5% bovine serum albumin (BSA, Sigma-Aldrich) in Tris-buffered saline with 0.05% Tween 20 (TBST; Sigma Aldrich) and incubated with the selected antibody overnight. Antibody details are as follows: anti-mouse NLRP3 (A41812012, AdipoGen Life Sciences), anti-mouse IL-1β (BAF401, R&D Systems), anti-mouse caspase-1 (clone Casper-1, AG-20B-0042-C100, AdipoGen Life Sciences), and anti-mouse α-tubulin (clone YL1/2; Abcam). Membranes were probed with the appropriate secondary antibody and visualized on the Bio-Rad ChemiDoc MP Imaging System via fluorescence.

### Quantification of cytokines and neutrophil elastase activity in BAL fluid

IL-1β and IL-18 were measured in BAL fluid by ELISA according to the manufacturer’s instructions (R&D Systems). Levels of IFNγ, IL-6, IL-10, IL-12p70, CCL-2/MCP-1, and TNF were quantified by cytokine bead array (BD mouse inflammation kit, BD Biosciences).

Neutrophil elastase activity was quantified as previously described [31]. BAL fluid or serially diluted porcine neutrophil elastase (Sigma-Aldrich, DY4517-05) was incubated with an equal volume of substrate N-Methoxysuccinyl-Ala-Ala-Pro-Val p-nitroanilide (Sigma-Aldrich, M4765) at 37 °C for 60 min and read at 405 nm. Substrate was reconstituted in Methyl-2-pyrolidone (Sigma-Aldrich) and then diluted in 110 mM Tris HCl (pH 8.0) for a final concentration of 0.5 mM per well.

### Flow cytometry analysis of BAL cells

BAL cells were isolated from whole BAL via centrifugation. Cell pellets were treated with a red blood cell (RBC) lysis buffer (Sigma Aldrich) for 5 min. Cells were resuspended in FACS buffer (PBS supplemented with 2% (v/v) FBS and 2 mM EDTA) to quench the RBC lysis buffer reaction. BAL cells were incubated with specific fluorescently labeled antibodies at 4 °C for 30 min in the presence of Fc receptor-blocking monoclonal antibodies against CD16/32 (clone 93, Thermo Fisher Scientific) to limit nonspecific antibody binding. BAL cells were stained with the following antibodies: CD11c (clone HL3, BD Biosciences), Ly6C (clone AL-21, BD Biosciences), CD64 (clone X54-5/7.1, BioLegend), CD3ε (clone 145-2C11, BioLegend), NK1.1 (clone PK136, BioLegend), Ly6G (clone 1A8, BD Biosciences), CD11b (clone M1/70, BioLegend), CD24 (clone M1/69, BioLegend), I-A^b^ (clone AF6-120.1, BD Biosciences) and Siglec-F (clone E50-2440, BD Biosciences). Viability was demonstrated using Zombie Aqua (BioLegend). Total live cells (viability dye^−^), neutrophils (Ly6G^+^ Ly6C^int^ SSC^lo^), natural killer (NK) cells (NK1.1^+^ CD3^−^), T cells (NK1.1^−^ CD3^+^), inflammatory macrophages (IM; Ly6G^−^ Ly6C^hi^), alveolar macrophages (AM; CD11c^+^ Siglec-F^+^ Ly6C^int^), dendritic cells (DCs; CD11c^+^ I-A^b+^) and eosinophils (CD24^+^ Siglec-F^+^ Ly6G^−^) were quantified by flow cytometry using an Aurora flow cytometer (Cytek Biosciences) and FlowJo™ 10 analysis software (BD Biosciences). Cell counts were determined using a standardized quantity of calibration particles/beads (ProSciTech) on a hemocytometer.

### Lung histopathology

Formalin-fixed and inflated lung tissues were incubated in 10% NBF overnight, processed, and embedded in paraffin wax. Each tissue block was serially sectioned (4 μm thickness) and stained with hematoxylin & eosin (H&E) or Masson’s trichrome. Separate unstained sections were imaged with multiphoton microscopy.

For morphometric analysis, Masson’s trichrome-stained sections were analyzed for alveolitis and fibrosis, and H&E-stained sections were analyzed for silicotic nodules. Masson’s trichrome-stained lung sections were graded for alveolitis on a subjective scale of 0 to 5 (0 = no inflammation, 1 = very mild, 2 = mild, 3 = moderate, 4 = marked, and 5 = severe inflammation), as previously described [32, 33]. Overall lung damage in Masson’s trichrome-stained lung sections was scored on a subjective scale of 0–4 using modified Ashcroft scoring criteria (0 = normal, 1 = minimal fibrous thickening of alveolar or bronchiolar walls, 2 = moderate thickening of walls without obvious change to lung architecture, 3 = increased fibrosis with definite damage to lung structure and formation of fibrous bands or small fibrous masses (including epithelial denudation) and presence of nodules, 4 = severe distortion of structure and large fibrous areas and total fibrous obliteration of the field). Sections were blinded and randomized, and samples corresponding to the least severe and most severe were assigned scores of 0 and 4/5, respectively, and five random fields per mouse were graded by three independent researchers. Lung sections were viewed on an Olympus BX60 microscope and photographed at 10x magnification for the Masson’s trichrome-stained sections and 20x or 4x magnification for H&E-stained sections with an Olympus DP74 color camera using Olympus cellSens Dimension software. Masson’s trichrome staining intensity was quantified using ImageJ software (positive pixel intensity per FOV). In addition, the silicotic nodule number, size/area, and cellularity were analyzed using Olympus cellSens Dimension software.

### Immunohistochemical and immunofluorescent imaging of lung tissue sections

For immunohistochemistry experiments, longitudinal paraffin-embedded lung tissue sections (4 μm) were dewaxed, rehydrated, and then incubated in 3% hydrogen peroxide to quench endogenous peroxidase activity. Heat-induced antigen retrieval was performed using a citrate buffer (10 mM citrate, pH 6 at 100 °C for 6 min). Slides were then blocked with CAS-Block Histochemical Reagent (Thermo Fisher Scientific) for 1 h before incubation with primary antibodies at 4 °C overnight: transforming growth factor β1 (TGFβ; mature secreted form, ab92486, Abcam) and α-smooth muscle actin (α-SMA, ab7817, Abcam). Incubation with secondary antibodies was performed at room temperature for 2 h. On separate slides, Terminal deoxynucleotidyl transferase-mediated dUDP nick-end labeling (TUNEL) assay was performed using the ApopTag Peroxidase In Situ Apoptosis Detection Kit (Merck Millipore) according to the manufacturer’s instructions. Sections were counterstained with hematoxylin and washed, and immunoreactivity was detected using diaminobenzidine (DAB) as the chromogen substrate (Agilent Dako, K3469). Slides were cover-slipped with D.P.X. mounting medium (Sigma Aldrich). Lung sections were visualized on an Olympus BX60 microscope, and five random FOVs per mouse were imaged at 40x magnification for TGFβ and α-SMA staining and 4x magnification for the TUNEL assay with an Olympus DP74 color camera using Olympus cellSens Dimension software. DAB staining (positive pixel count per FOV) was quantified using ImageJ software, as previously described [33].

For confocal imaging of lung tissue sections from NLRP3 reporter mice, paraffin-embedded sections were dewaxed and rehydrated. Slides were then washed with PBS and incubated with Hoechst 33342 nuclear staining (Thermo Fisher). Slides were mounted using Fluorescence Mounting Medium (Agilent) and imaged at 40x magnification using a Nikon A1R confocal microscope (Nikon, Japan) and processed using ImageJ software.

For immunofluorescence staining of lung tissue sections, paraffin-embedded sections were dewaxed and rehydrated. Heat-induced antigen retrieval was performed with citrate buffer (10 mM citrate, pH 6 at 100 °C for 6 min). Slides were then blocked with CAS-Block Histochemical Reagent (Thermo Fisher Scientific) for 1 h before incubation with an IL-17A primary antibody (PA579470, Thermo Fisher Scientific) at 4 °C overnight. Slides were then washed with PBS containing 0.01% Tween 20 and incubated with appropriate secondary antibodies (Thermo Fisher) for 1 h, followed by Hoechst 33342 nuclear staining (Thermo Fisher). Slides were finally mounted using Fluorescence Mounting Medium (Agilent) and imaged at 40x magnification using a Nikon A1R confocal microscope and processed using ImageJ software. The % positive cells per FOV was analyzed using ImageJ software.

### Culture and in vitro stimulation of bone marrow-derived macrophages

Bone marrow cells from wildtype and NLRP3 reporter mice were differentiated for 7 days in DMEM supplemented with 10% (v/v) FCS and 1% (v/v) penicillin/streptomycin solution and M-CSF (20% (v/v) L929 mouse fibroblast supernatant). Differentiated bone marrow–derived macrophages (BMDMs) were seeded at 1 × 10^5^ cells/96-well (ELISA, LDH) or 5 × 10^5^ cells/12-well (immunoblot) 24 h prior to experimentation. Cells were incubated with LPS (O114:B5; 100 ng/mL, InvivoGen) for 3 h and then stimulated with NLRP3 inflammasome activators silica (250 µg/mL; Min-U-Sil-5, U.S. Silica), nigericin (10 µM; InvivoGen), ATP (5 mM; Sigma-Aldrich) or poly(dA: dT) (200 ng; InvivoGen) transfected with Lipofectamine 2000 (Life Technologies). After a further 6 h, cell supernatants and cell lysates were collected. Levels of IL-1β were quantified by ELISA according to the manufacturer’s instructions (R&D Systems). Cell supernatants were assayed for levels of lactate dehydrogenase (LDH) using a CytoTox 96 Non-radioactive Cytotoxicity Assay (Promega).

### Statistical analysis

Data were tested for normality and analyzed by GraphPad Prism Version 10 software (Graphstats Technologies, India). Student’s *t*-test (two-tailed, unpaired) was used when comparing two values. When comparing three or more sets of values, a One-way analysis of variance (ANOVA) was used with either Tukey’s or Dunnett’s multiple comparisons post-hoc test. A *P* value of < 0.05 was considered statistically significant.

## Results

### Silica particles penetrate the distal lung following intranasal delivery, resulting in the formation of silicotic nodules and fibrosis

**Fig. 1:**
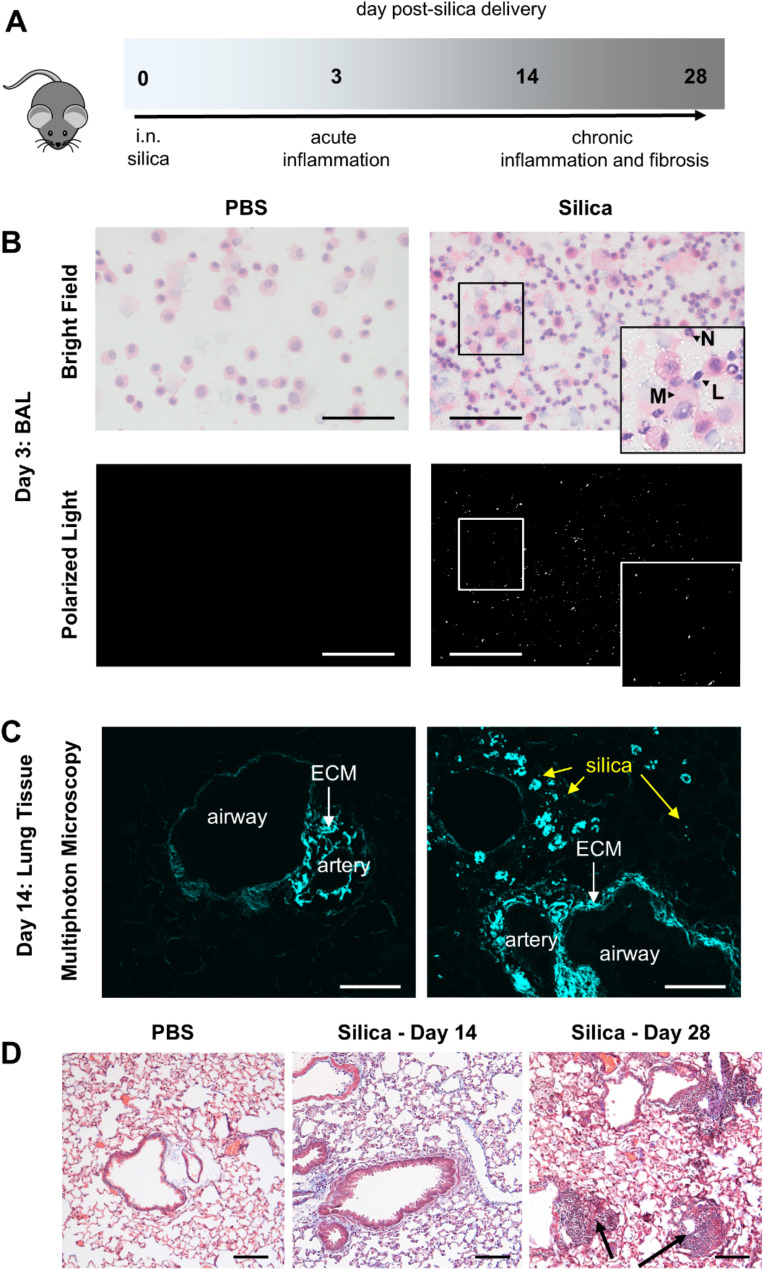
Silica particles persist in the lung, causing chronic inflammation, nodule formation, and fibrosis. (**A**-**D**) Groups of 5 C57BL/6J mice were intranasally administered 2 mg of silica. Control mice received PBS alone. Data are representative of at least 2 independent experiments. (**A**) Schematic of disease progression following silica delivery. (**B**) Cytospin analysis of H&E-stained bronchoalveolar lavage (BAL) at day 3. Representative brightfield images (top panels). Arrows indicate the presence of immune cells with morphology resembling neutrophils (N), lymphocytes (L), and macrophages (M) cells. The boxed area demonstrates macrophages containing silica crystals, providing a magnified view. Silica particles in the same field of view were additionally visualized under polarized light (bottom panels). 400x magnification (scale bar 100 μm). (**C**) Visualization of silica at day 14 in formalin-fixed lung tissue sections using multiphoton microscopy. 25x magnification (scale bar 100 μm). Yellow arrows indicate silica. Extracellular matrix (ECM), airways, and arteries are indicated. (**D**) Representative images of Masson’s trichrome-stained, formalin-fixed lung tissue sections at day 14 and 28. 40x magnification (scale bar 100 μm). Gross histological changes characteristic of silicosis were observed. Silicotic nodules are indicated by the arrows

Short-term exposure to a bolus of silica dust can lead to the development of acute silicosis or silicoproteinosis, which is characterized by severe alveolitis and alveolar proteinosis [8]. To model silicosis, mice were administered 2 mg of silica via the intranasal route. Acute inflammation was analyzed on day 3, as well as chronic inflammation and fibrosis on days 14 and 28 (Fig. 1A). Mice were weighed 3 times a week but did not display any clinical signs of disease, including reduced mobility and significant weight loss, up to 28 days post-silica delivery (Supplementary Fig. 1). Cytospin analysis of H&E-stained cells recovered from the BAL at day 3 indicated that intranasal delivery of silica increased overall airway cellularity (Fig. 1B). Notably, neutrophils were frequently observed in silica but not PBS-treated mice. Under brightfield microscopy, silica particles were identified within macrophages. Visualization of BAL under polarized light also confirmed the presence of ‘free’ silica particles (i.e., not cell-associated). Consistent with the inability to clear silica, analysis of lung tissue sections using multiphoton microscopy indicated silica particles remained distributed around the airways on day 14 and throughout the parenchyma (Fig. 1B). Collagen fibers, which can also be observed under multiphoton microscopy, were increased around the airways and pulmonary arteries by day 14 (Fig. 1C), indicative of greater extracellular matrix (ECM) deposition. By day 28, key features of silicosis were evident in Masson’s trichrome-stained tissue sections. This included hallmark silicotic nodule formation, airway thickening, damage to the lung structure, and increased detection of collagen fibers (Fig. 1D). Of note and in line with other studies [14, 27, 34], intranasal inoculation of mice with a lower dose of silica (0.5 or 1 mg) did not result in silicotic nodule formation (data not shown). Collectively, these results demonstrate the establishment of a progressive mouse model of silica-induced persistent inflammation, fibrosis, and nodule formation.

### Silica promotes NLRP3 expression and activation in the lung

**Fig. 2:**
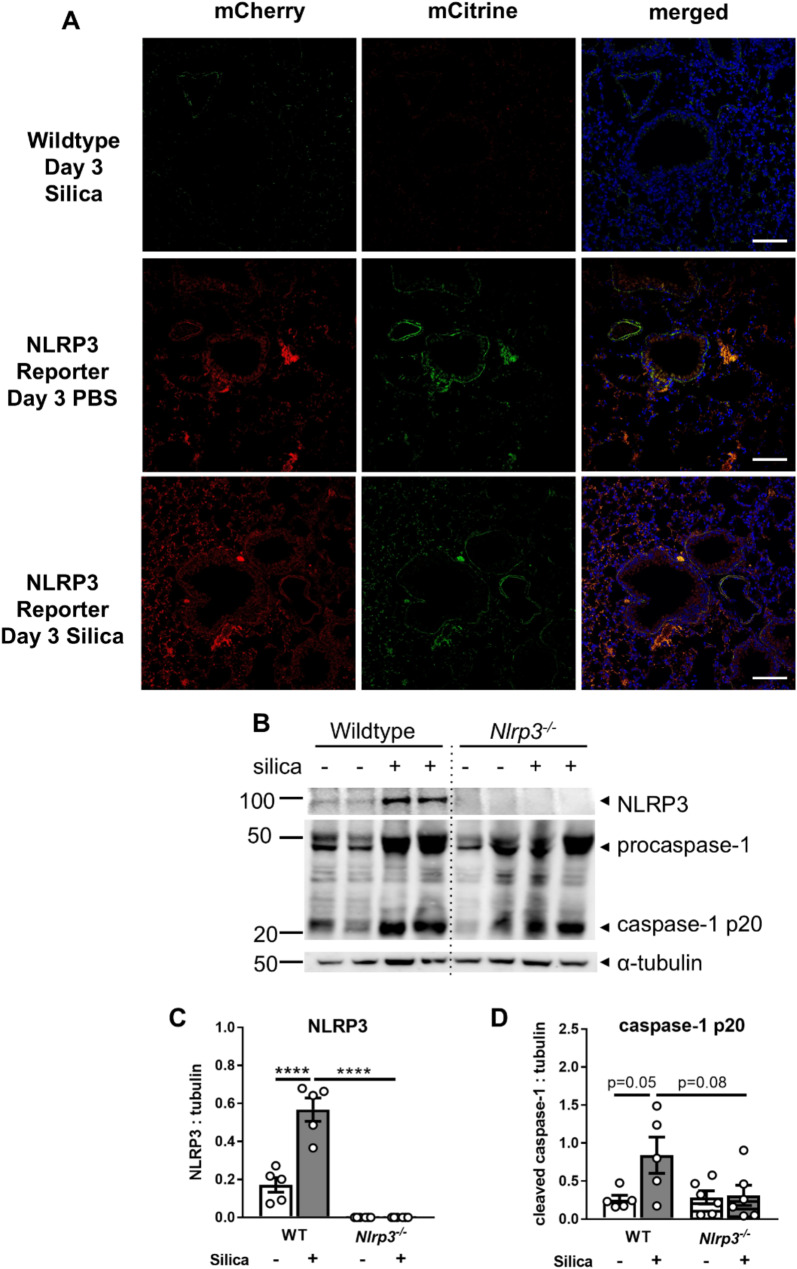
Silica drives the expression and activation of NLRP3 in the lung. (**A**) Reporter mice expressing mCitrine (green) and mCherry (red) at distal junctions of NLRP3 under the native promoter were intranasally administered 2 mg of silica. Control reporter mice received PBS alone. Wildtype (WT) mice were also included as a control. *n* = 5 per group. On day 3, NLRP3 expression in lung tissue sections was examined by confocal microscopy. Merged images show colocalization of mCitrine and mCherry with Hoechst (blue) at 40x magnification (scale bar 100 μm). (**B**-**D**) *Nlrp3*^−/−^ mice and WT littermates were intranasally administered 2 mg of silica. Control mice received PBS alone. (**B**) Immunoblot of NLRP3, procaspase-1 (p45), cleaved caspase-1 (p20), and tubulin proteins in lung tissue lysates. Expression of (**C**) NLRP3 and (**D**) cleaved caspase-1 (p20) relative to tubulin. (**C**-**D**) Data are presented as mean ± SEM, with each data point representing an individual animal. *n* = 5–6 per group. ****P < 0.0001, One-way ANOVA. Data are pooled from 3 independent experiments

A limited number of studies have implicated a role for the NLRP3 inflammasome in silicosis [14, 27, 35], however, the distribution of NLRP3 expression and its activation in the lung are currently not well characterized. We generated a novel reporter mouse (NLRP3-CHCI) that expresses mCitrine and mCherry at the N- and C-termini of NLRP3, respectively (Supplementary Fig. 2A), enabling real-time tracking of NLRP3 expression and localization in the lung. The reporter is placed under the native NLRP3 promoter, ensuring that the expression pattern reflects physiological regulation and not artificial overexpression. Importantly, macrophages generated from these mice respond to NLRP3 agonists such as nigericin, adenosine triphosphate (ATP), and silica, secreting IL-1β, undergoing cell death, and maturing caspase-1 and IL-1β (Supplementary Fig. 2B-E) commensurate to wildtype macrophages, indicating the fluorescent tags do not interfere with inflammasome function. To examine NLRP3 expression in the lung, wildtype and NLRP3 reporter mice were administered 2 mg silica or PBS alone via the intranasal route, and lung tissues were formalin-fixed and inflated 3 days later. mCherry and mCitrine expression in lung tissue sections were visualized by confocal imaging. Silica delivery increased the expression of mCherry and mCitrine, particularly in the alveoli, indicating greater NLRP3 expression throughout the lung (Fig. 2A).

To better understand the role of the NLRP3 inflammasome, wildtype and *Nlrp3*^*−/−*^ mice were exposed to silica via the intranasal route. Immunoblot analysis indicated silica delivery increased the expression of NLRP3 and procaspase-1 in the lung at day 3 in wildtype mice (Fig. 2B-D). Increased expression of the mature p20 subunit of caspase-1, which is indicative of inflammasome activation, was observed in the lungs of wildtype mice at day 3 post-silica delivery, with a trend for reduced expression in *Nlrp3*^*−/−*^ mice (*P* = 0.08). Taken together, these results show silica delivery upregulates NLRP3 expression and leads to NLRP3 activation in the lung.

### NLRP3 deficiency limits silica-induced production of IL-1β and IL-18 in the airways

**Fig. 3:**
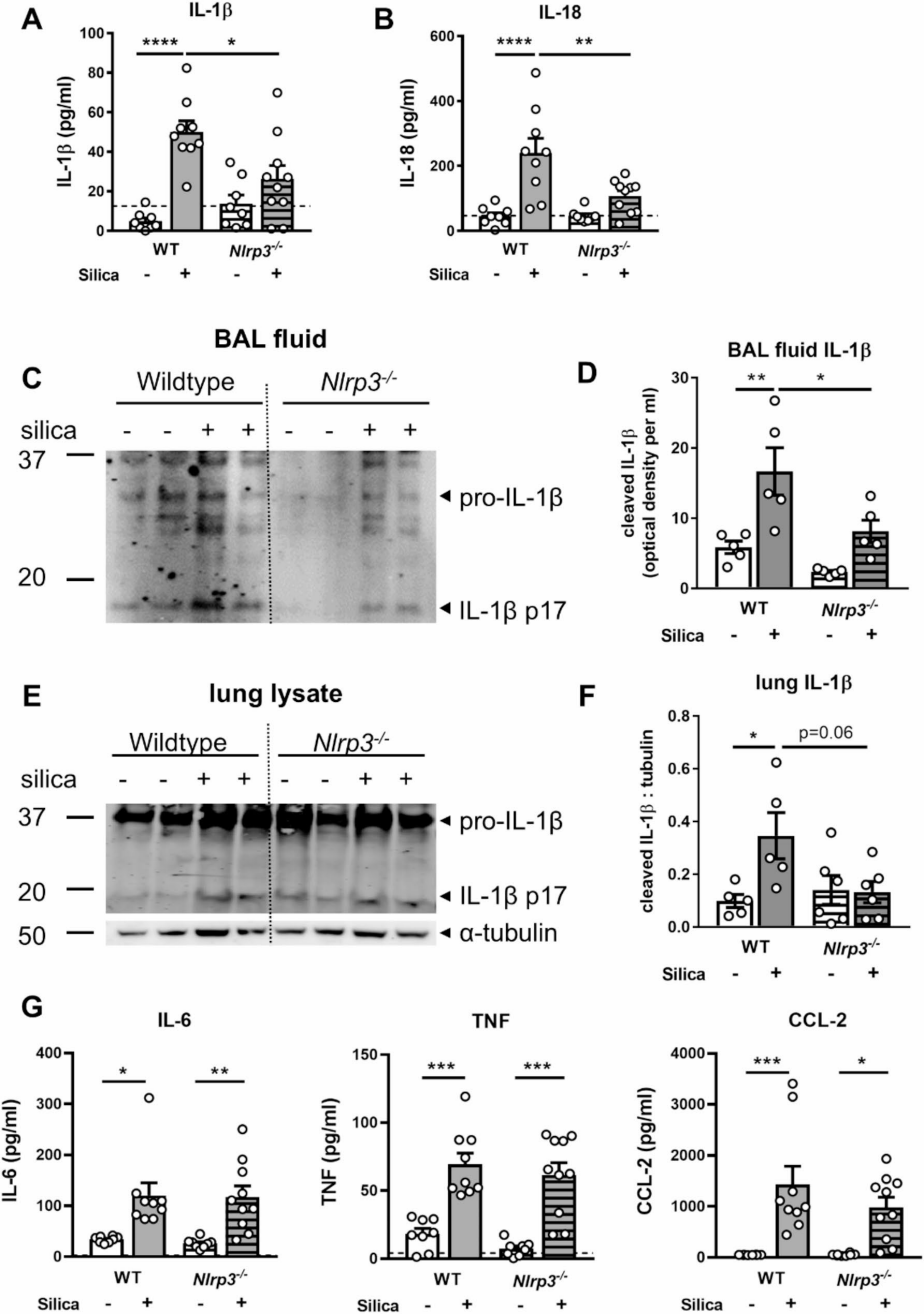
NLRP3 deficiency limits silica-induced IL-1β and IL-18 responses in the lung. (**A**-**G**) *Nlrp3*^−/−^ mice and wildtype (WT) littermates were intranasally administered 2 mg of silica. Control mice received PBS alone. Analysis was performed on day 3. Levels of (**A**) IL-1β and (**B**) IL-18 in BAL fluid, determined by ELISA. (**C**) Immunoblot of equivalent volume of concentrated BAL fluid for pro-IL-1β (p31) and cleaved IL-1β (p17). (**D**) IL-1β p17 expression represented as optical density per mL of BAL fluid. (**E**) Immunoblot of lung tissue lysates for pro-IL-1β (p31), IL-1β (p17), and tubulin protein. (**F**) IL-1β (p17) expression in lung tissues relative to tubulin. (**G**) Levels of IL-6, TNF, and CCL-2 in BAL fluid determined by cytokine bead array. (**A**-**G**) Data are presented as mean ± SEM, with each data point representing an individual animal. *n* = 5–10 per group. *P < 0.05, **P < 0.01, ***P < 0.001, ****P < 0.0001, One-way ANOVA. Data are pooled from 3 independent experiments. The detection limit of each assay is noted as a dotted line

IL-1β and IL-18 are potent pro-inflammatory cytokines that are potential mediators of silica-induced inflammation. Caspase-1 within an active NLRP3 inflammasome cleaves the cytokine precursors pro-IL-1β and pro-IL-18 into their bioactive and secretory forms, IL-1β and IL-18, respectively [1]. Levels of IL-1β and IL-18 in BAL fluid determined by ELISA were halved in *Nlrp3*^*−/−*^ mice compared to wildtype controls at day 3 following silica exposure (Fig. 3A-B). Immunoblot analysis confirmed silica upregulated the expression of the mature p17 subunit of IL-1β in BAL fluid from wildtype mice (Fig. 3C-D), which is indicative of inflammasome activation. Importantly, NLRP3 deficiency correlated with significantly reduced expression of the cleaved p17 subunit of IL-1β in the airways. In addition, immunoblot analysis of lung tissue demonstrated silica increased the expression of the mature p17 subunit of IL-1β at day 3 in wildtype but not NLRP3-deficient mice (Fig. 3E-F). While the cytokines IL-6, TNF, and CCL-2 were all increased in BAL fluid following silica exposure, levels remained unchanged with NLRP3 deletion (Fig. 3G). Together, these findings suggest that following silica delivery, NLRP3 inflammasome activity promotes maturation of IL-1β and IL-18 in the lung.

### NLRP3 deficiency limits the infiltration of conventional and Siglec-F^+^ neutrophils into airways following silica delivery

**Fig. 4:**
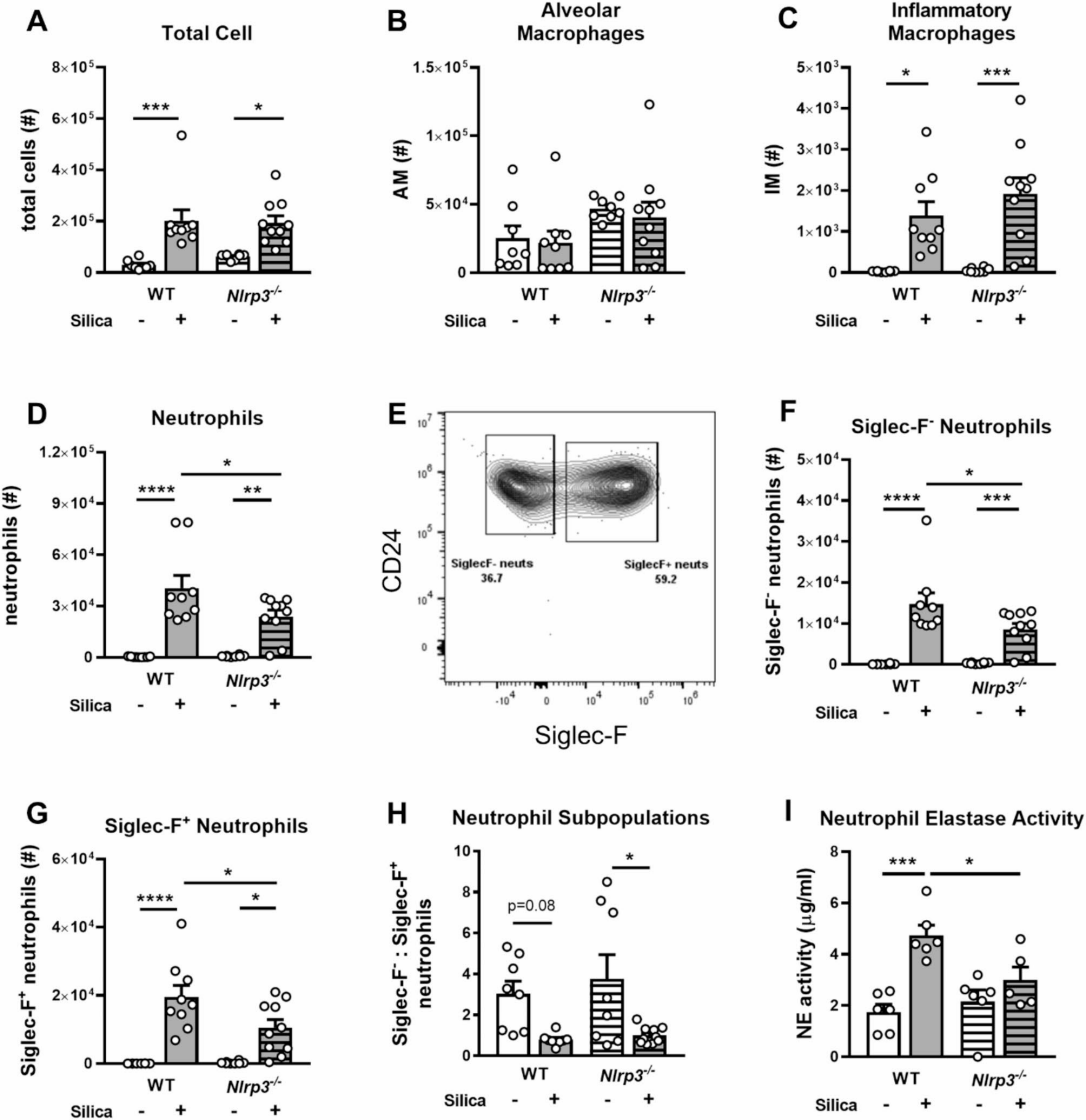
NLRP3 deficiency diminishes silica-induced neutrophil responses in the lung. (**A**-**I**) *Nlrp3*^−/−^ mice and wildtype (WT) littermates were intranasally administered 2 mg of silica. Control mice received PBS alone. Numbers (#) of (**A**) total live cells, (**B**) alveolar macrophages (AMs), (**C**) Ly6C^hi^ inflammatory macrophages (IMs), and (**D**) Ly6G^*+*^ neutrophils in the BAL on day 3, determined by flow cytometry. (**E**) Representative flow cytometry gating strategy for Siglec-F^−^ and Siglec-F^+^ neutrophil subpopulations. Numbers (#) of live (**F**) Siglec-F^−^ and (**G**) Siglec-F^+^ neutrophils. (**H**) Ratio of Siglec-F^−^ to Siglec-F^+^ neutrophils. (**I**) Neutrophil elastase (NE) activity in BAL fluid. The detection limit of the assay was 12.5 pg/mL. (**A**-**I**) Data are presented as mean ± SEM, with each data point representing an individual animal. *n* = 6–10 per group. Data are pooled from 3 independent experiments. *P < 0.05, **P < 0.01, ***P < 0.001, ****P < 0.0001, One-way ANOVA

Having initially established that silica induces the early infiltration of immune cells in the airways (Fig. 1B), we investigated whether NLRP3 deficiency alters the innate immune cell composition of the airways. Total cells, infiltrating neutrophils, inflammatory monocytes/macrophages (IM), natural killer (NK) cells, dendritic cells (DCs), eosinophils, and T cells, as well as resident alveolar macrophages (AM) in the BAL, were enumerated at day 3 post-silica by flow cytometry (Supplementary Fig. 3). Silica administration increased total airway cellularity (Fig. 4A), particularly infiltrating Ly6C^hi^ IMs (Fig. 4C) and Ly6G^+^ neutrophils (Fig. 4D). Deletion of NLRP3 markedly reduced total neutrophil numbers (Fig. 4D) but had no impact on the recruitment of IMs (Fig. 4C). There was no significant change in the number of resident AMs following silica treatment (Fig. 4B). Numbers of DCs, eosinophils, NK cells, and T cells were also not altered by NLRP3 deficiency at day 3 (Supplementary Fig. 4A-D).

Further flow cytometric analysis of Ly6G^+^ neutrophils in the airways revealed the presence of two distinct subpopulations (Fig. 4E). These included conventional Siglec-F^−^ neutrophils, which contribute to the inflammatory response, as well as Siglec-F^+^ neutrophils, which are reported to be long-lived and involved in profibrotic processes, including aberrant wound healing under disease conditions [36]. As Siglec-F can also be expressed on murine eosinophils, we performed additional cytospin-based analysis of BAL cells to confirm they morphologically resemble neutrophils (Supplementary Fig. 4E/F). Of note, in wildtype mice, Ly6G^+^ Siglec-F^+^ neutrophils represented ~ 50–60% of neutrophils on day 3. Total numbers of Siglec-F^−^ and Siglec-F^+^ neutrophils were significantly reduced in the airways of NLRP3-deficient mice (Fig. 4F-G). Notably, the ratio of Siglec-F^−^ to Siglec-F^+^ neutrophils trended lower in silica-treated wildtype mice compared to PBS-treated controls (Fig. 4H). This ratio remained consistent in NLRP3-deficient mice, suggesting that NLRP3 is critical for the early infiltration of Siglec-F^−^ and Siglec-F^+^ neutrophils. Importantly, neutrophils are the major source of neutrophil elastase, which is known to contribute to tissue damage in silicosis [37]. Consistent with these previous observations, silica treatment increased neutrophil elastase activity in BAL fluid at day 3 in wildtype mice (Fig. 4I). Critically, the reduced neutrophil numbers observed in NLRP3-deficient mice correlated with a significant reduction in neutrophil elastase activity in the airways.

Lastly, IL-17A-producing cells, such as γδ T cells and T helper 17 (Th17) cells, have been implicated in driving silicosis [38]. We therefore quantified IL-17A^+^ cells present in lung tissue sections at day 28 via confocal microscopy. NLRP3 deficiency had no impact on the frequency of IL-17A^+^ cells within the airways (Supplementary Fig. 5A-C). Additionally, no difference was seen in the presence of IL-17A^+^ cells within silicotic nodules (Supplementary Fig. 5D).

Together, these results demonstrate NLRP3 promotes the infiltration of conventional and a unique population of Siglec-F-expressing neutrophils, which impacts fibrotic-associated airway elastase activity.

### NLRP3 deficiency reduces silica-induced lung fibrosis, nodule size, and damage

**Fig. 5:**
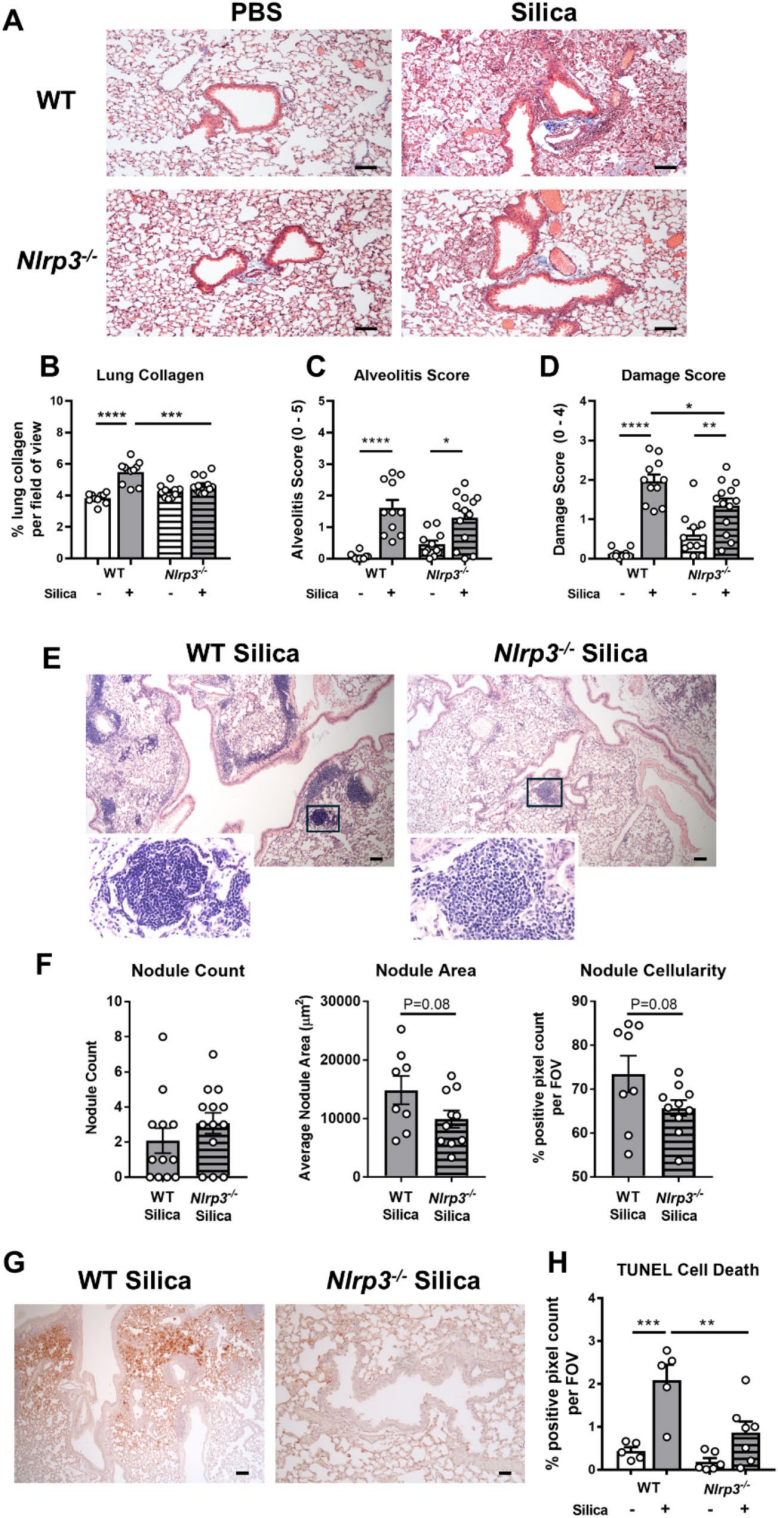
Mice deficient in NLRP3 display reduced silica-induced lung damage and fibrosis. (**A**-**H**) *Nlrp3*^−/−^ mice and wildtype (WT) littermates were intranasally administered 2 mg of silica. Control mice received PBS alone. On day 28, lung tissues were formalin-fixed and inflated. Histological analysis of Masson’s trichrome-stained lung tissue sections was performed. (**A**) Representative images at 10x magnification. Scale bar 100 μm. Lung sections were randomized, blinded, and analyzed or scored for (**B**) collagen deposition (Masson’s trichrome staining intensity per field of view (FOV)), (**C**) alveolitis (scale 0–5), and (**D**) lung damage (scale 0–4). (**E**) Representative images of H&E-stained lung tissue sections, imaged at 4x magnification. Scale bar 100 μm. The boxes on these images indicate the areas that are magnified to demonstrate silicotic nodules determined with Olympus cellSens Dimension software. Lung sections were randomized and analyzed for nodule (**F**) total count, average size (µm^2^), and cellularity (% positive pixel count per FOV). (**G**) Representative images at 4x magnification of TUNEL assay labeling of cell death in lung tissue sections. Scale bar 100 μm. (**H**) Quantification of TUNEL staining determined with ImageJ software. Data are presented as the mean percentage positive pixel count per FOV. (**A**-**H**) Data are presented as mean ± SEM, with each data point representing an individual animal. *n* = 5–13 per group. Data are pooled from 3 independent experiments. *P < 0.05, **P < 0.01, ***P < 0.001, ****P < 0.0001, One-way ANOVA

Silicosis is characterized by irreversible lung tissue damage driven by the excessive collagen deposition and severe alveolitis. Masson’s trichrome staining of lung sections from silica-treated mice revealed extensive tissue damage with localized collagen accumulation around airways, as well as increased alveolar and lung inflammation by day 28 (Fig. 5A-D). Deletion of NLRP3 reduced the severity of these gross pathological features, implicating a role for NLRP3 in the chronic phase of silicosis.

A hallmark of silicosis is the formation of silicotic nodules [11], which are granulomatous structures composed of clusters of inflammatory cells and fibrotic fibers. A potential role for NLRP3 in driving the formation of these nodules has not been explored. Quantification of H&E-stained lung tissue sections demonstrated a trend toward reduced nodule size and cellularity in NLRP3-deficient mice compared to wildtype controls (Fig. 5E-F). NLRP3 activation results in pyroptosis, a lytic form of cell death [39]. Additionally, nonresolving inflammation can promote tissue injury. To assess the impact of NLRP3 deficiency on cell death, TUNEL labeling was performed on lung tissue sections. *Nlrp3*^−/−^ mice displayed reduced tissue and epithelial cell death relative to wildtype littermates (Fig. 5G-H), further supporting a detrimental role for NLRP3. Together these indicate a previously unrecognized role for NLRP3 in the formation of silicotic nodules, while reinforcing its established role in silica-induced pulmonary immunopathology and fibrosis.

### NLRP3 deficiency reduces silica-induced expression of TGFβ and α-SMA in the lung

**Fig. 6:**
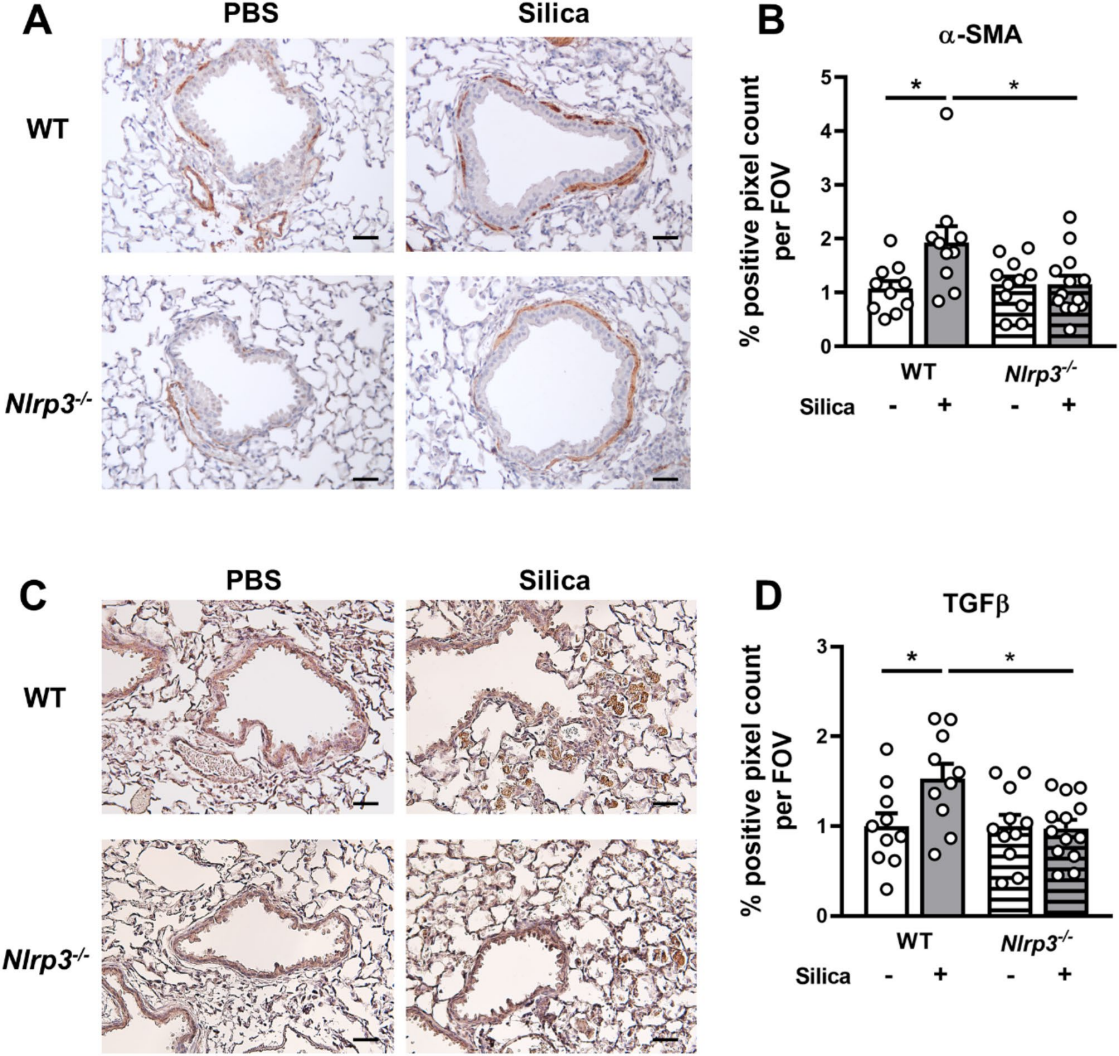
NLRP3 deficiency limits the expression of TGFβ and α-SMA in the lung following silica delivery. (**A**-**D**) *Nlrp3*^−/−^ mice and wildtype (WT) littermates were intranasally administered 2 mg of silica. Control mice received PBS alone. On day 28, lung tissues were formalin-fixed and inflated. (**A**) Representative images at 20x magnification of immunohistochemical analysis of α-SMA in lung tissue sections. Scale bar 100 μm. (**B**) Quantification of α-SMA expression determined with ImageJ software. Data are presented as the mean percentage positive pixel count per FOV. (**C**) Representative images at 20x magnification of immunohistochemical analysis of TGFβ (mature/active form) in lung tissue sections. Scale bar 20 μm. (**D**) Quantification of TGFβ expression determined with ImageJ software. Data are presented as the mean percentage positive pixel count per FOV. (**A**-**D**) Data are presented as mean ± SEM, with each data point representing an individual animal. *n* = 10–13 per group. Data are pooled from 3 independent experiments. *P < 0.05, **P < 0.01, ***P < 0.001, ****P < 0.0001, One-way ANOVA

Since alterations in lung tissue damage, including the formation of silicotic nodules, had been confirmed in NLRP3-deficient mice (Fig. 5), we next investigated potential downstream mechanisms driving these fibrotic processes. Myofibroblasts are contractile, fibrogenic cells that are primary ECM-secreting cells [40]. Examination of lung sections revealed that silica-induced α-SMA expression, a marker of myofibroblast activation, was reduced in NLRP3-deficient mice (Fig. 6A-B). Additionally, the profibrotic cytokine TGFβ plays a key role in driving epithelial-mesenchymal transition (EMT), a process in which epithelial cells adopt a mesenchymal phenotype. This transition contributes to the deposition of ECM components, including collagen, which is the primary driver of lung tissue remodeling [40, 41]. Immunohistochemical staining of lung tissue sections was performed at day 28 to assess expression of the mature/active form of TGFβ (Fig. 6C-D). Silica delivery increased the expression of TGFβ specifically around airways and in inflammatory cells in the lung parenchyma, with a greater effect observed in wildtype mice compared to NLRP3-deficient mice. Together these results demonstrate for the first time that NLRP3 inflammasome responses play a critical role in driving TGFβ-mediated myofibroblast activation and fibrosis in response to silica-induced lung injury.

## Discussion

Silicosis remains a prevalent global burden, affecting millions of workers annually [2]. Despite the devastating outcomes of silicosis, there is a lack of effective therapeutic options available [7]. To identify new therapeutic strategies, we need to better understand the pathogenesis of silicosis. The NLRP3 inflammasome is an innate immune sensor that was discovered in 2004 [42] and is now well described to be activated by disruptions in cellular homeostasis [43]. NLRP3 activation is tightly regulated to prevent excessive inflammation and to ensure that the immune response is appropriate without becoming dysregulated [14]. Early in vitro studies identified silica activates the NLRP3 inflammasome in macrophages [14, 44], leading to the secretion of potent inflammatory cytokines and plasma membrane rupture [14, 35, 45]. More recently, limited studies have implicated an in vivo role for NLRP3 in promoting lung pathology following silica exposure [26, 27]. Here, we provide mechanistic insights into the role of NLRP3 in driving silica-induced pulmonary disease, further highlighting its potential as a therapeutic target. Specifically, we identified NLRP3 drives early silica-induced inflammation in the airways, including the production of potent cytokines IL-1β and IL-18, as well as conventional and fibrotic neutrophil responses. NLRP3 deficiency limited subsequent pulmonary damage and fibrosis, including silicotic nodule formation at a more advanced stage of silicosis. This reduced immunopathology correlated with diminished expression of the profibrotic cytokine TGFβ, as well as myofibroblast activation, fundamental events that drive fibrosis.

Silica exposure has been shown to increase NLRP3 expression as well as NLRP3-associated cytokines, including IL-1β, in lung epithelial cells [46, 47] and mouse and human macrophages [14, 27, 48]. However, these studies are limited to in vitro, cell-based assessments, leaving critical in vivo mechanisms largely unexplored. NLRP3 expression is well described to be driven by NFκB responses [49]. Consistent with this, immunoblot analysis of lung tissues revealed silica delivery upregulated the expression of NLRP3 within 3 days (Fig. 2). Our attempts to examine NLRP3 expression in lung tissue sections via confocal staining were unsuccessful due to non-specific staining with multiple commercially available antibodies, in tissues from *Nlrp3*^*−/−*^ mice. Critically, using a novel NLRP3 reporter controlled under the native promoter, we identified that NLRP3 expression is rapidly increased diffusely throughout the lung following silica delivery. Of note, NLRP3 was observed in epithelial cells lining the alveoli, suggesting that its expression is not leukocyte-specific and suggests that epithelial NLRP3 may play a role in driving silicosis pathology. Consistent with activation of NLRP3, silica treatment led to the cleavage and activation of caspase-1 (Fig. 2). There was also a trend toward reduced caspase-1 activation in the absence of NLRP3, further confirming NLRP3 activation following silica exposure.

Caspase-1, within an active NLRP3 inflammasome complex, drives the cleavage and maturation of potent pro-inflammatory cytokines, IL-1β and IL-18. Levels of these cytokines in the airways were markedly reduced in NLRP3-deficient mice (Fig. 3). However, responses were not completely abrogated, suggesting they may be matured by an NLRP3-independent pathway. Silica-induced release of cytoplasmic DNA from damaged or dead cells [50] could potentially be sensed by absent in melanoma 2 (AIM2) inflammasomes [51, 52], however, this has not been fully investigated in vivo. IL-1β and IL-18 can also be cleaved by other enzymes, including cathepsin-controlled proteases such as neutrophil elastase [53]. Interestingly, we did observe reduced neutrophil elastase activity in NLRP3-deficient mice (Fig. 4), suggesting fewer neutrophil-derived proteases would be available to process IL-1β and IL-18, which likely contributes to the partial reduction in IL-1β and IL-18 levels due to reduced cleavage of pro-IL-1β and pro-IL-18. Other mechanisms may therefore compensate for the loss of these cytokines in the absence of NLRP3.

NLRP3-dependent IL-1β and IL-18 responses are crucial for enhancing inflammatory responses by promoting the recruitment and activation of immune cells such as neutrophils [54]. NLRP3 deficiency reduced numbers of Ly6G^+^ neutrophils in the airways, the key first responders (Fig. 4). These findings are consistent with our recent studies demonstrating that treatment of mice with the NLRP3 and NLRP1 dual inhibitor reduces silica-induced infiltration of neutrophils [55]. Additionally, our results implicating a major role for NLRP3 in driving neutrophil responses are also consistent with studies involving inhibition of the NLRP3 inflammasome [56] or IL-1β [57] itself in other lung disease models. Critically, we describe here for the first time that silica drives the early infiltration of two distinct subpopulations of Siglec-F^−^ and Siglec-F^*+*^ neutrophils into the airways, which may have unique roles in silicosis disease progression (Fig. 4). Mice deficient in NLRP3 displayed reduced numbers of Siglec-F^−^ and Siglec-F^+^ neutrophils, and this correlated with reduced levels of activated neutrophil elastase activity in the BAL. Conventional Siglec-F^−^ neutrophils are traditionally short-lived cells involved in inflammatory processes. Siglec-F^+^ neutrophils have been described as long-lived cells that accumulate and have an activated phenotype in comparison to Siglec-F^−^ neutrophils in lung and nasal mucosa models of cancer [58] and allergic rhinitis [59], respectively. Interestingly, Siglec-F^+^ neutrophils have been shown to be essential for creating a profibrotic microenvironment in the kidney and lung [36, 60] producing a greater amount of pro-inflammatory and pro-fibrotic markers including IL-1β, TGFβ, collagen I, and α-SMA [36]. Tissue damage has been shown to trigger the transition of conventional Siglec-F^−^ neutrophils to Siglec-F^+^ neutrophils [60]. Shin et al. further demonstrated that the upregulation of Siglec-F^+^ neutrophils was dependent on the production of ATP and subsequent activation of the P2X1 receptor in a model of air-pollutant-induced asthma exacerbations [60]. Interestingly, ATP is a well-characterized activator of NLRP3. Inhibition of the P2 X1 receptor has been shown to reduce Siglec-F-expressing neutrophils [60], suggesting a potential therapeutic target for mitigating fibrosis. Taken together, these findings warrant further investigation of neutrophil activity in silicosis and the role of these cells in disease progression. Understanding the specific contributions of these different neutrophil subpopulations could provide insights into targeting neutrophil-mediated inflammation in silicosis and other fibrotic lung diseases.

Silicosis progression involves an interplay between inflammation and aberrant tissue repair processes [61]. Our findings demonstrate that NLRP3 deficiency not only reduces early inflammatory cytokine production but also attenuates lung damage, cell death, and fibrosis (Fig. 5). These results correlated with reduced expression of profibrotic markers, including mature TGFβ and α-SMA (Fig. 6). In the lung, TGFβ is a key regulator of fibrosis [62], promoting the differentiation of fibroblasts into myofibroblasts, which are the primary producers of ECM proteins, including collagen. TGFβ is produced by many cells, including macrophages and lymphocytes [40]. The reduction in α-SMA, a hallmark of myofibroblast activation [40], suggests that NLRP3 signaling contributes to the activation of fibroblasts and subsequent tissue remodeling. In mouse models of silicosis, elevated and persistent expression of IL-1β precedes the development of fibrosis, suggesting a critical role for IL-1β in disease progression. Evidence suggests IL-1β promotes Th17, TGFβ, and platelet-derived growth factor responses, which contribute to ongoing disease. IL-18 has also been shown to induce EMT in a model of bleomycin-induced pulmonary fibrosis [63, 64]. Interestingly, NLRP3 deficiency reduced early IL-1β and IL-18 responses (Fig. 3) but did not appear to play a key role in augmenting Th17 responses (Supplementary Fig. 5). Importantly, NLRP3 deficiency limits the size of silicotic nodules and overall lung fibrosis, further highlighting the contributions of NLRP3 in promoting hallmark features of silicosis. The protective effects of NLRP3 deficiency extend beyond reducing inflammatory and fibrotic mediators, as evidenced by decreased overall lung damage. This finding suggests that the NLRP3 inflammasome may directly influence cellular responses by increasing IL-1β and IL-18 production in response to silica particles in both immune and structural cell populations. Silica-induced cell death and the release of cellular danger signals (i.e., DAMPs), which could also drive NF-κB and TGFβ responses, thus promoting IL-1β and fibrotic responses [1]. In line with this, it has been reported that neutralization of IL-1ɑ in the lung following silica delivery limits the production of IL-1β in the airways [65]. Future studies investigating cell-type-specific contributions of NLRP3, particularly in epithelial and mesenchymal cells, could provide valuable insights into mechanisms underlying inflammasome activation and fibrosis progression.

Therapeutic strategies targeting the NLRP3 inflammasome, either through direct inhibitors or by modulating its upstream activators, may hold promise for preventing or treating silicosis. Studies assessing the efficacy of anti-NLRP3 or anti-IL-1β treatments following chronic exposure to silica have demonstrated the role of NLRP3 activation in the progression of advanced disease. Treatment with an anti-IL-1β monoclonal antibody, the IL-1 receptor antagonist anakinra [38, 66], and the plant alkaloid, tetrandrine [48], an inhibitor of NLRP3 responses, reduced the levels of inflammatory cells and cytokines in BAL from silica-exposed mice. Notably, inhibiting NLRP3 or IL-1β signaling has been shown to slow disease progression and reduce the severity of lung fibrosis by reducing collagen deposition and TGFβ activity [48]. Targeting multiple pathways, including the NLRP3 inflammasome, as a therapeutic approach may be critical to suppress the overstimulation of inflammatory pathways in silicosis.

Overall, these findings highlight for the first time the potential of inflammasome-related markers, such as NLRP3 expression levels and inflammasome-associated cytokines, as diagnostic or prognostic biomarkers for silicosis. Critically, neutrophil elastase activity could serve as a surrogate marker for disease severity, given its role in cytokine maturation and tissue damage. It is, however, important to note that this study involved one single silica inhalation event, which does not fully reproduce the reiterated inhalation of an exposed worker. Furthermore, species-specific differences may produce different results in human inhalation outcomes as compared to mice. Targeting the NLRP3 inflammasome, either through specific inhibitors or broader anti-inflammatories, may represent promising therapeutic strategies. Combining these strategies with existing antifibrotics could further enhance treatment efficacy and improve overall patient outcomes.

## Electronic supplementary material

Below is the link to the electronic supplementary material.

Supplementary Material 1

## Data Availability

Data is provided within the manuscript or supplementary information files.

## Abbreviations

α-SMA: Alpha smooth muscle actin
AM: Alveolar macrophage
BAL: Bronchoalveolar lavage
DAMPs: Damage-associated molecular patterns
ECM: Extracellular matrix
EMT: Epithelial-mesenchymal transition
IL-1β: Interleukin 1 beta
IL-18: Interleukin 18
IM: Inflammatory macrophages/monocyte
NLRP3: NOD-, LRR- and pyrin domain-containing protein 3
NLRP3-CHCI: NLRP3 reporter mouse
TGFβ: Transforming growth factor beta
