# Supplementary material for: NLRP3 deficiency abrogates silica-induced neutrophil infiltration, pulmonary damage and fibrosis
Source: Respir Res. 2025 Mar 21;26:109. doi: 10.1186/s12931-025-03192-y (PMC11929224; doi:10.1186/s12931-025-03192-y)

SUPPLEMENTARY MATERIAL

NLRP3 deficiency abrogates silica-induced neutrophil infiltration, pulmonary damage, and fibrosis

Maggie Lam[1,2], Kristian Barry[1,2], Christopher J Hodges[1,2], Christopher M Harpur[1], James D Ong[1,2], Sarah Rosli[1,2], Alison West[1,2], Lovisa Dousha[3,4], Paul J Hertzog[1,2], Ashley Mansell[1,2,5*], and Michelle D Tate[1,2*#]

1. Centre for Innate Immunity and Infectious Disease, Hudson Institute of Medical Research, Clayton, Victoria, 3168, Australia.
2. Department of Molecular Translational Sciences, Monash University, Clayton, Victoria, 3186, Australia.
3. Monash Lung, Sleep, Allergy, and Immunology, Monash Medical Centre, 246 Clayton Rd, Clayton, Melbourne, Victoria, 3168, Australia.
4. Department of Medicine, Monash University, Clayton, Melbourne, Victoria, 3168, Australia.
5. Department of Microbiology, Anatomy, Physiology, and Pharmacology, La Trobe University, Bundoora, Victoria, 3086, Australia.

* authors contributed equally.

#Correspondence: Michelle D. Tate; Address: Centre for Innate Immunity and Infectious Diseases, Hudson Institute of Medical Research, 27-31 Wright Street, Clayton, Victoria, 3168, Australia. Email: michelle.tate@hudson.org.au.

SUPPLEMENTARY FIGURES

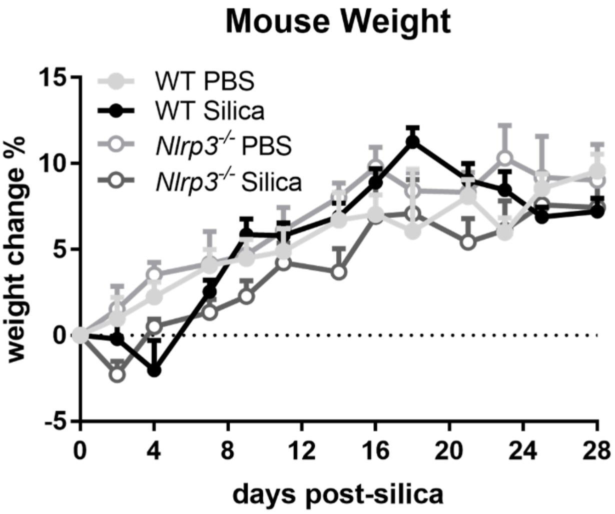

Supplementary Figure 1. Silica delivery does not result in significant changes in body weight. *Nlrp3*$^{-/-}$ mice and wildtype (WT) littermates were intranasally administered 2 mg of silica. Control mice received PBS alone. Mice were weighed over a 28-day period. Results are expressed as mean percent weight change ± SEM. *n* = 4-6 per group.

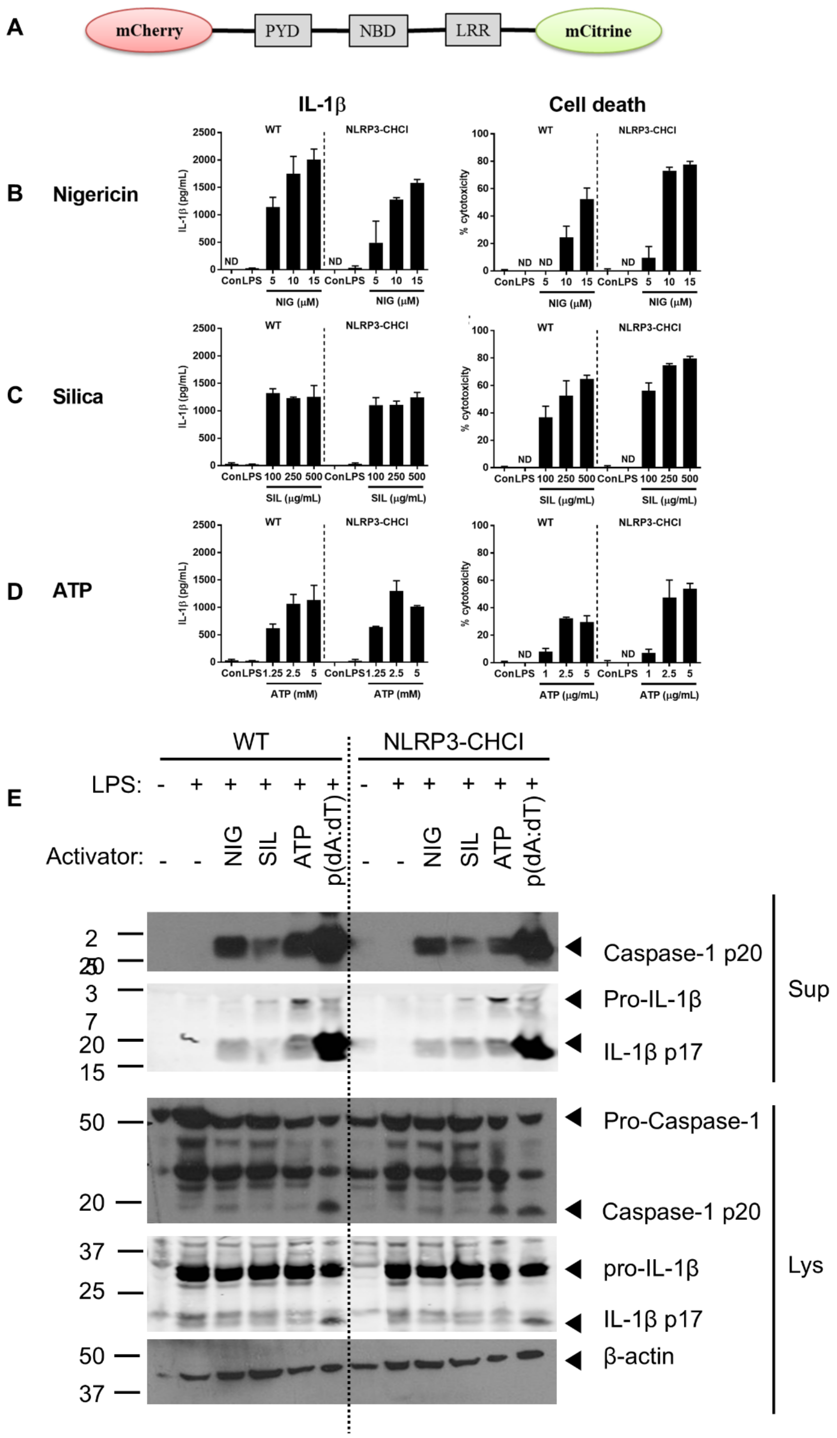

Supplementary Figure 2. NLRP3 reporter macrophages respond to NLRP3 agonists *in vitro*. Reporter mice (NLRP3-CHCI) expressing mCitrine (green) and mCherry (red) at distal junctions of NLRP3 under the native promoter were generated as described in the Methods. **(A)** Schematic of the NLRP3 reporter. NBD = nucleotide-binding domain, LRR = leucine-rich repeats, PYD = pyrin domain. **(B-D)** Primary bone marrow-derived macrophages were cultured from wildtype (WT) C57BL/6J and NLRP3-CHCI reporter mice, treated with LPS (100 ng/mL) in triplicate for 3 h, and then stimulated with nigericin (NIG; 10 µM), silica (SIL; 250

μg/mL), or ATP (5 mM) for 6 h. Poly(dA:dT) (200 ng) was included as a control. Cell-free supernatants were harvested, and levels of **(B)** IL-1β and **(C)** LDH release/cell death were determined by ELISA or colorimetric assay, respectively. **(B-D)** Data are presented as mean ± SEM and are representative of three independent experiments. **(E)** Immunoblot of cell lysate (Lys) and supernatants (Sup) for pro-IL-1β (p31), IL-1β (p17), pro-caspase-1 (p45), cleaved caspase-1 (p20), and β-actin protein. Data are representative of three independent experiments. Arrows indicate the bands of interest.

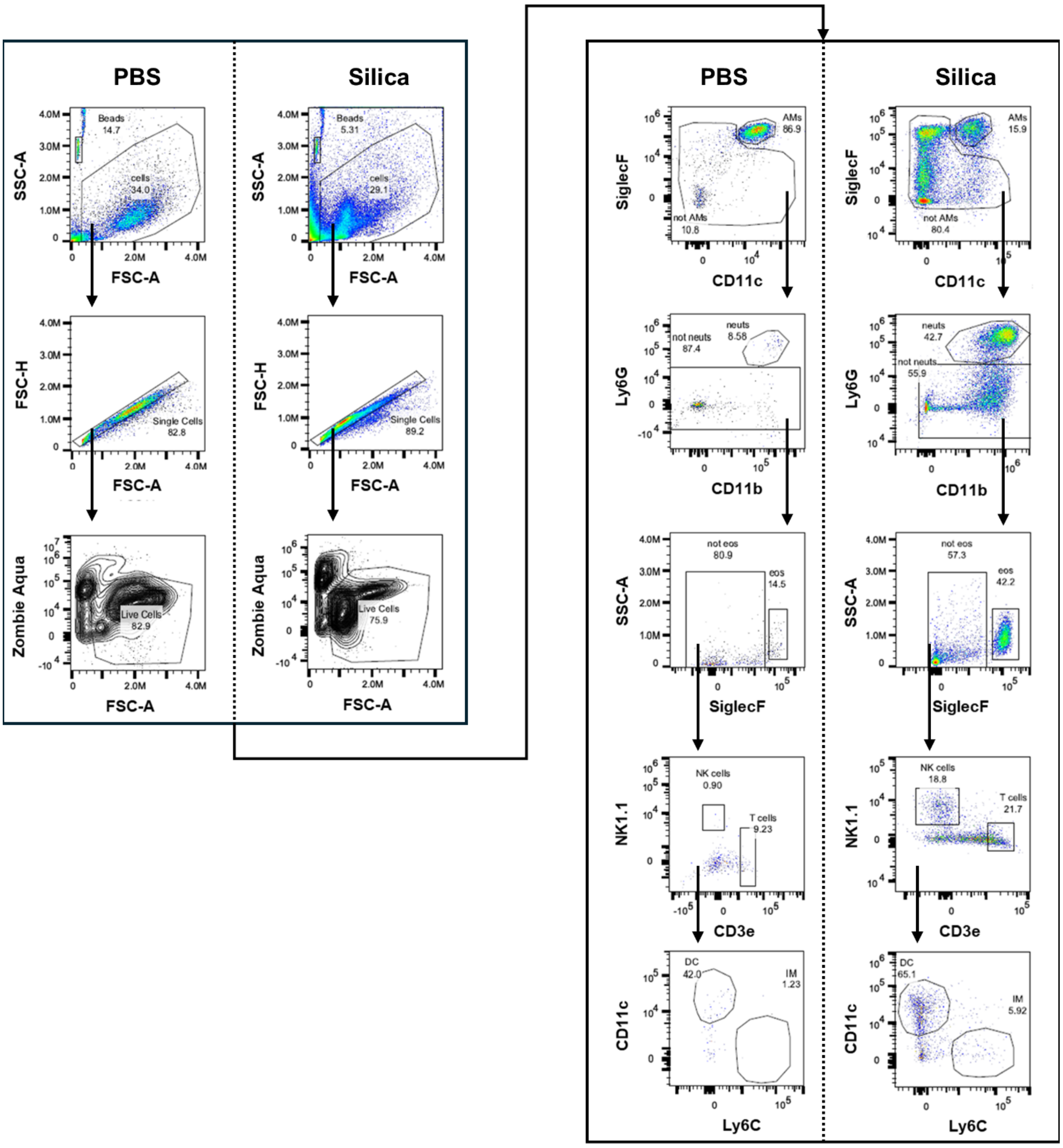

Supplementary Figure 3. Flow cytometry gating strategy for analysis of BAL cells. Mice were intranasally administered 2 mg of silica, and BAL was performed on day 3. Control mice received PBS alone. Representative flow cytometry gating strategy for alveolar macrophages (AM), neutrophils (neuts), eosinophils (eos), inflammatory macrophages (IMs), dendritic cells (DC), natural killer (NK) cells, and T cells. Calibration beads were utilized to enumerate cell numbers.

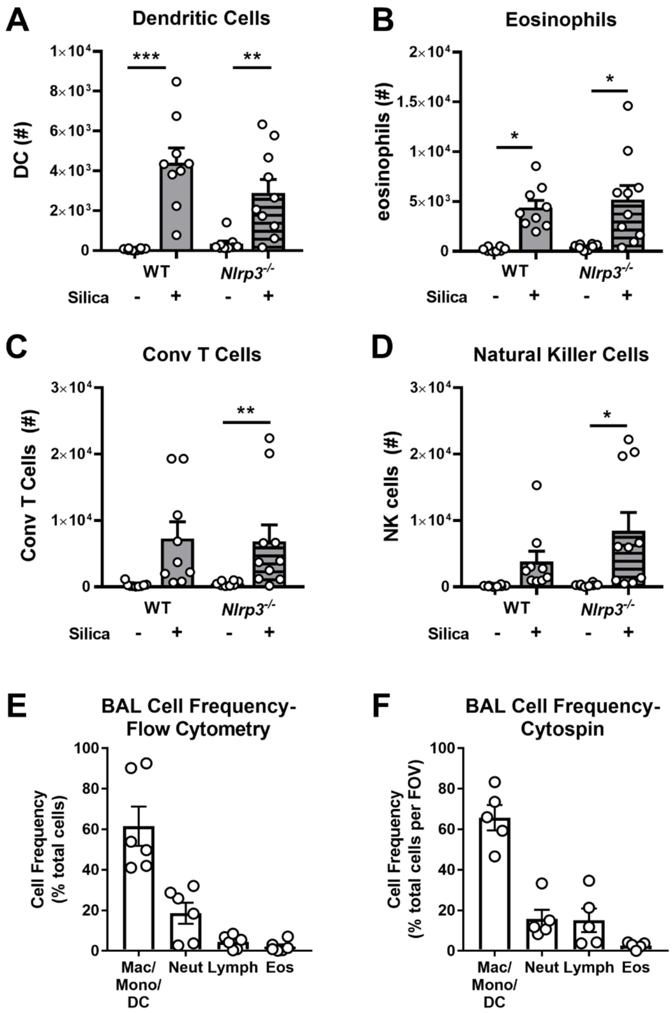

Supplementary Figure 4. NLRP3 deficiency does not alter dendritic cell, eosinophil, T cell, and natural killer cell numbers in the airways. (A-F) *Nlrp3*$^{-/-}$ mice and wildtype (WT) littermates were intranasally administered 2 mg of silica, and BAL was performed on day 3. Control mice received PBS alone. Numbers (#) of live (Zombie Aqua viability dye^{-}) **(A)** dendritic cells (DC), **(B)** eosinophils, **(C)** conventional (conv) T cells, and **(D)** natural killer (NK) cells, determined by flow cytometry. **(A-D)** Data are presented as mean ± SEM, with each data point representing an individual animal. *n* = 5-10 per group. Data are pooled from 2 independent experiments. **(E/F)** WT mice were intranasally administered 2 mg of silica. On day 3, flow cytometric **(E)** and cytospin-based **(F)** analysis of BAL cells was performed to enumerate macrophages (mac)/monocytes (mono)/DCs, neutrophils (Neut), lymphocytes (Lymph), and eosinophils (Eos). Data are presented as mean % ± SEM, with each data point representing an individual animal. *n* = 5-6 per group.

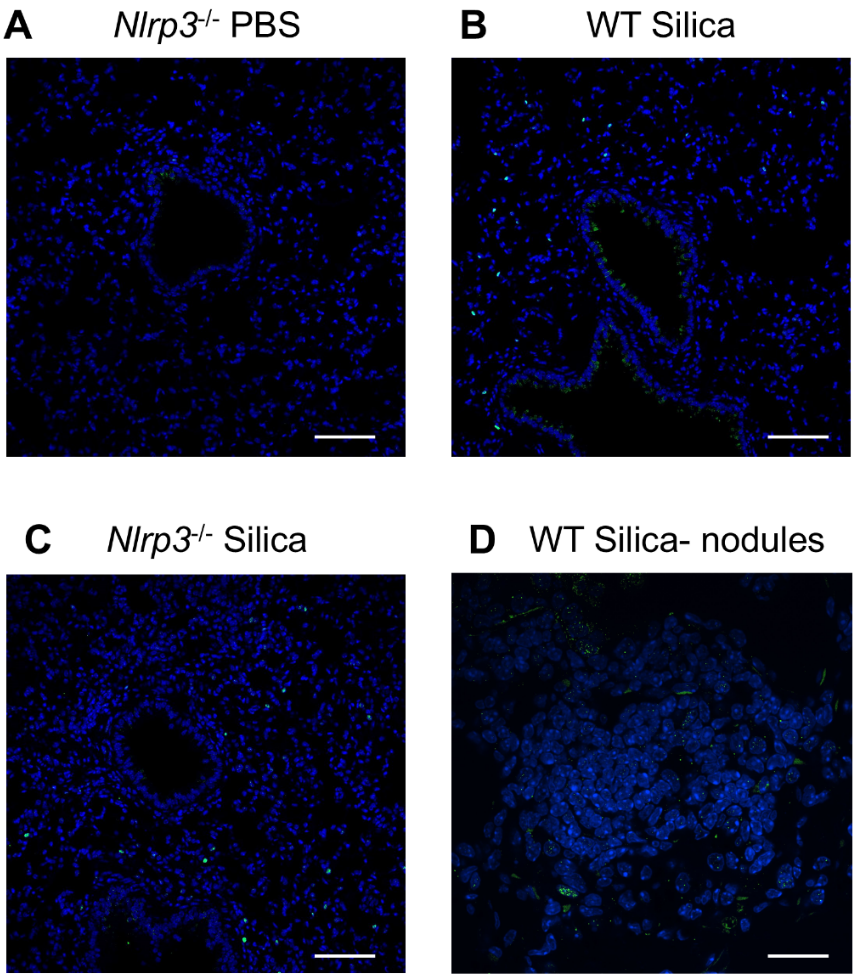

Supplementary Figure 5. NLRP3 deficiency does not alter IL-17A⁺ cell numbers in the lung. (B-D) *Nlrp3*$^{-/-}$ mice and wildtype littermates were intranasally administered 2 mg of silica. **(A)** Control mice received PBS alone. **(A-D)** On day 28, lung tissues were formalin-fixed and inflated. IL-17A-expressing cells in lung tissue sections were examined by confocal microscopy. Representative images at 40x magnification (scale bar 100 μm).

Figure 2 Full Blots

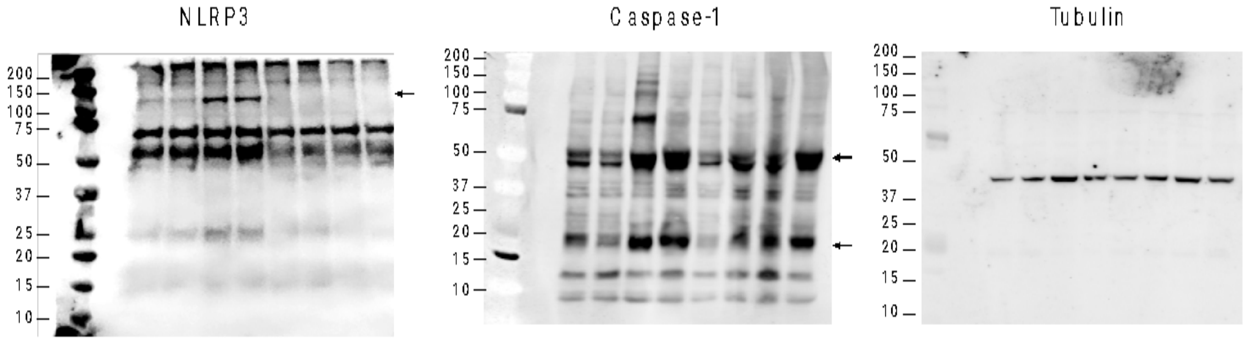

Figure 3C Full Blots

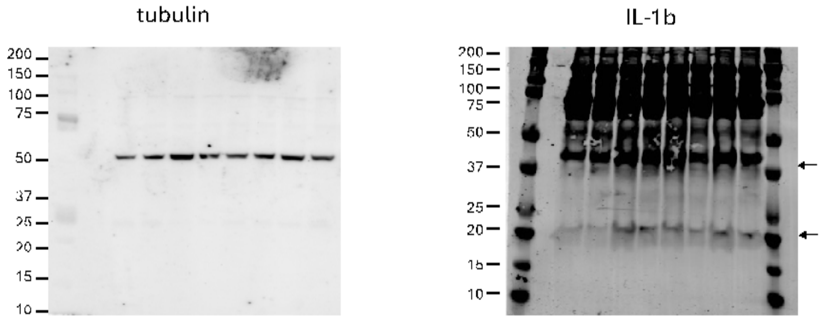

Figure 3E Full Blot

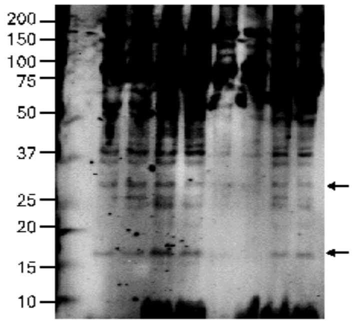

Supplement: Supplementary file 1 — Supplementary Material 1 [file 12931_2025_3192_MOESM1_ESM.pdf]
